# Case report of Salmonella derby septicemia complicated with co-occurrence of disseminated intravascular coagulation and thrombotic microangiopathy

**DOI:** 10.1186/s12879-022-07913-2

**Published:** 2022-12-07

**Authors:** Yingxin Lin, Lei Huang, Yunliang Tu, Bin Huang, Sheng Zhang, Yingqun Chen, Weijia Li

**Affiliations:** Department of Intensive Care, Peking University Shenzhen Hospital, Shenzhen, China

**Keywords:** Case report, Co-occurrence, Thrombotic microangiopathy, Disseminated intravascular coagulation, Thrombocytopenia

## Abstract

**Background:**

Both disseminated intravascular coagulation and thrombotic microangiopathy are complications of sepsis as Salmonella septicemia, respectively. They are related and have similar clinical characteristics as thrombopenia and organ dysfunctions. They rarely co-occur in some specific cases, which requires a clear distinction.

**Case presentation:**

A 22-year-old woman had just undergone intracranial surgery and suffered from Salmonella derby septicemia with multiorgan involvement in the hospital. Laboratory workup demonstrated coagulation disorder, hemolytic anemia, thrombocytopenia, and acute kidney injury, leading to the co-occurrence of disseminated intravascular coagulation and secondary thrombotic microangiopathy. She received antibiotics, plasma exchange therapy, dialysis, mechanical ventilation, fluids, and vasopressors and gained full recovery without complications.

**Conclusion:**

Disseminated intravascular coagulation and secondary thrombotic microangiopathy can co-occur in Salmonella derby septicemia. They should be treated cautiously in diagnosis and differential diagnosis. Thrombotic microangiopathy should not be missed just because of the diagnosis of disseminated intravascular coagulation. Proper and timely identification of thrombotic microangiopathy with a diagnostic algorithm is essential for appropriate treatment and better outcomes.

**Supplementary Information:**

The online version contains supplementary material available at 10.1186/s12879-022-07913-2.

## Background

Both thrombotic microangiopathy (TMA) and disseminated intravascular coagulation (DIC) are acute life-threatening conditions. They share similar clinical presentations as a bleeding tendency, thrombocytopenia, and organ failure, which makes it challenging to distinguish one from the other [1].

DIC is typically featured by the simultaneous occurrence of widespread vascular clot deposition, compromising blood supply to various organs and contributing to organ failure [2–6]. The characteristic of DIC is systemic activation of the coagulation system, followed by consumption of platelets and clotting factors and secondary fibrinolysis arising from both inflammatory and non-inflammatory causes [7]. The generation of fibrin-related markers (FRMs) in DIC reflects microvascular changes [1]. Though there is no gold standard and no specific biomarker for diagnosing DIC, a reliable diagnosis of DIC can be made through simple scoring algorithms based on readily available routine hemostatic parameters [6]. About 35% of cases of severe sepsis with Gram-negative and Gram-positive microorganisms may be complicated by DIC [6, 8–10].

TMA is not as common as DIC. According to the experts’ experience, physicians encounter an average of three cases of TMA per year in the intensive care unit (ICU) [11]. TMA is a pathological term describing small vessel (arterioles and capillaries) injury and microvascular thrombosis. It is defined by microangiopathic hemolytic anemia (MAHA), thrombocytopenia, and organ failure of the kidney, central nervous system, and other organs [1, 12, 13]. It includes Shiga toxin-associated hemolytic uremic syndrome (STEC-HUS), atypical hemolytic uremic syndrome (aHUS), and thrombotic thrombocytopenic purpura (TTP), as well as secondary TMA with a coexisting diseases/condition (e.g., infection, malignancy, autoimmune disease, pregnancy, transplantation, or drug) [11, 14, 15]. Core processes of TMA is the remarkable activation, aggregation, and consumption of platelet originating from widespread inflammation and vascular endothelial cell injuries [11, 14, 15].

Prompt identification and accurate etiological diagnosis are crucial for early therapeutic approaches to minimize organ damage and improve patient survival. For differential diagnosis, the elevation of FRMs is required in DIC [15], deficiency of A disintegrin-like and metalloprotease with thrombospondin type 1 motifs 13(ADAMTS13) activity (< 10%) is required in TTP, Shiga toxin is required in STEC-HUS, abnormalities of the complement system are necessary for aHUS [10, 13, 14]. Several diagnostic algorithms that aid rapid differential diagnosis have been published [16–19]. Only a few have been tailored to intensivists [11, 20], and none have mentioned the co-occurrence of DIC and TMA.

The co-occurrence of DIC and TMA in the sepsis population is extremely rare and should raise our attention. Herein we first report a Salmonella derby septicemia complicated with DIC and secondary TMA in a 22-year-old woman. Comprehensive management with antibiotics, plasma exchange, and supportive therapies resulted in good outcomes without chronic sequelae.

## Case presentation

A 22-year-old female patient underwent craniopharyngioma surgery in our hospital. She developed panhypopituitarism postoperatively, supplemented with desmopressin, hydrocortisone, and L-thyroxine. She was otherwise healthy, and her familial medical histories were negative for renal and thrombotic diseases.

On the 20th postoperative day, she developed a fever(38.2℃), vomiting, and non-bloody diarrhea with exposure to undercook seafood in the neurosurgical ward. She was transferred to ICU for respiratory distress the next day.

On the 21st postoperative day, her condition deteriorated. She presented tachycardia (145/min), hypotension (80/65 mmHg), tachypnea (40/min) on a high-flow nasal cannula, and anuric acute kidney injury (AKI). Physical examination revealed non-palpable skin petechiae, subcutaneous hematoma in the right inguinal area, and diffuse bilateral lung crackles.

Laboratory test results in ICU were as follows (Table 1). Her hematology revealed anemia (68 g/L) and thrombocytopenia (6 × 10^9^/L). Coagulation suggested prolonged activated partial thromboplastin time (50.2 s, reference 28.0–43.0 s) and prothrombin time (21.0 s, reference 11.0–15.0 s), elevated fibrinogen and fibrin degradation products (FDPs) (70.84 mg/L, reference 0–5.00 mg/L). Urinalysis showed proteinuria. Urinary sediment contained 3–5 white blood cells and > 60 red blood cells per high-power field. Procalcitonin was 96.33 ng/mL. The renal function test showed increased urea nitrogen (10.9 mmol/L) and serum creatinine (161 umol/L). Chest computed tomography revealed bilateral pulmonary filtrations and pleural effusions. Cardiac echography revealed moderate tricuspid regurgitation with estimated pulmonary systolic pressure of 43 mmHg.

**Table 1:** Laboratory parameters

Variables	On presentation of DIC	On presentation of TMA	Reference range
WBC (×10^9^/L)	10.60	10.22	3.5–9.5
Hb(g/L)	68	60	115–150
PLT(×10^9^/L)	6	5	125–350
RET (%)	–	2.42	0.5–2.00
Schistocytes (%)	–	5	
LDH (IU/L)	–	2794	120–246
ALT(IU/L)	28	107	9–66
Indirect bilirubin(umol/L)	20.9	29.6	3.1–23
Creatinine (umol/L)	116	155*	42–96
BUN (umol/L)	8.87	12.11*	2.5–7.1
PT(s)	21.0	14.6	11.00–15.00
PT–INR	1.82	1.09	0.80–1.20
APTT (s)	50.20	35.5	28.0–43.0
FDPs (g/L)	70.84	–	0–5.00
Fibrinogen (g/L)	2.54	5.20	2.00–4.00
PCT (ng/mL)	> 100	> 100	< 0.05

*WBC* white blood cell, *Hb* hemoglobin, *PLT* platelet, *RET* reticulocyte count, *LDH* lactate dehydrogenase, *ALT* alanine aminotransferase, *BUN* blood urea nitrogen, *PT* prothrombin time, *s* second, *PT-INR* prothrombin time-international normalized ratio, *APTT* activated partial thromboplastin time, *FDP* fibrinogen and fibrin degradation products, *PCT* procalcitonin

*The patient was on continuous veno-venous hemodialysis

Her blood pressure coagulation disorder returned to normal on the 23rd postoperative day. She was still anuric. She was anemic despite transfusions of 10.5 U packed red blood cells in 3 days. Her hemoglobin was 60 g/L. Her platelet count was 21 × 10^9^/L. Lactate dehydrogenase (LDH) was 2794 IU/L. The reticulocyte count was 2.42%; The percentage of schistocytes was 5%. All the above revealed Coombs’ negative microangiopathic hemolytic anemia with thrombocytopenia, indicating TMA.

The results of the etiological workup were as follows (Table 2; Additional file 1: Table S1). Complement factors (C3, C4, CH50), immunoglobulins, autoimmune, anti-phospholipid, and vasculitis antibodies all tested normal, except for a mild decrease of immunoglobulin G (6.68 g/L, reference 8.60–17.40 g/L). Further hematological tests returned negative, including CD59 of red blood cells and white blood cells, anti-platelet antibodies, and levels of ferritin, folic acid, and vitamin B12. Salmonella derby was isolated from blood cultures, while Escherichia coli and Enterococcus faecalis were isolated from urine cultures. Neither O-157 Escherichia coli nor Salmonella was isolated from stool and urine cultures. Mildly reduced ADAMTS13 activity was reported (46%; reference 70–120%). Complement Factor H was 517.4 ug/ml (reference 247.0–1010.8 ug/ml). Factor H autoantibody and inhibitor of ADAMTS13 were both negative.

**Table 2:** Laboratory workup of TMA etiology

Variables	Result	Reference range
C3 (g/L)	0.83	0.70–1.40
C4 (g/L)	0.11	0.10–0.40
CH50(U/mL)	30.3	23.0–46.0
ADAMTS13 activity–before plasma exchange (%)	46	70–120
ADAMTS13 activity–after plasma exchange (%)	86	70–120
ADAMTS13 inhibitor (BU)	0	0–0.6
Complement factor H antibody	Negative	Negative
Complement factor H (ug/ml)	517.4	247.0–1010.8

*CH50* 50% hemolytic unit of complement, *ADAMTS13* Plasma A disintegrin-like and metalloprotease with thrombospondin type 1 motifs 13

**Fig. 1:**
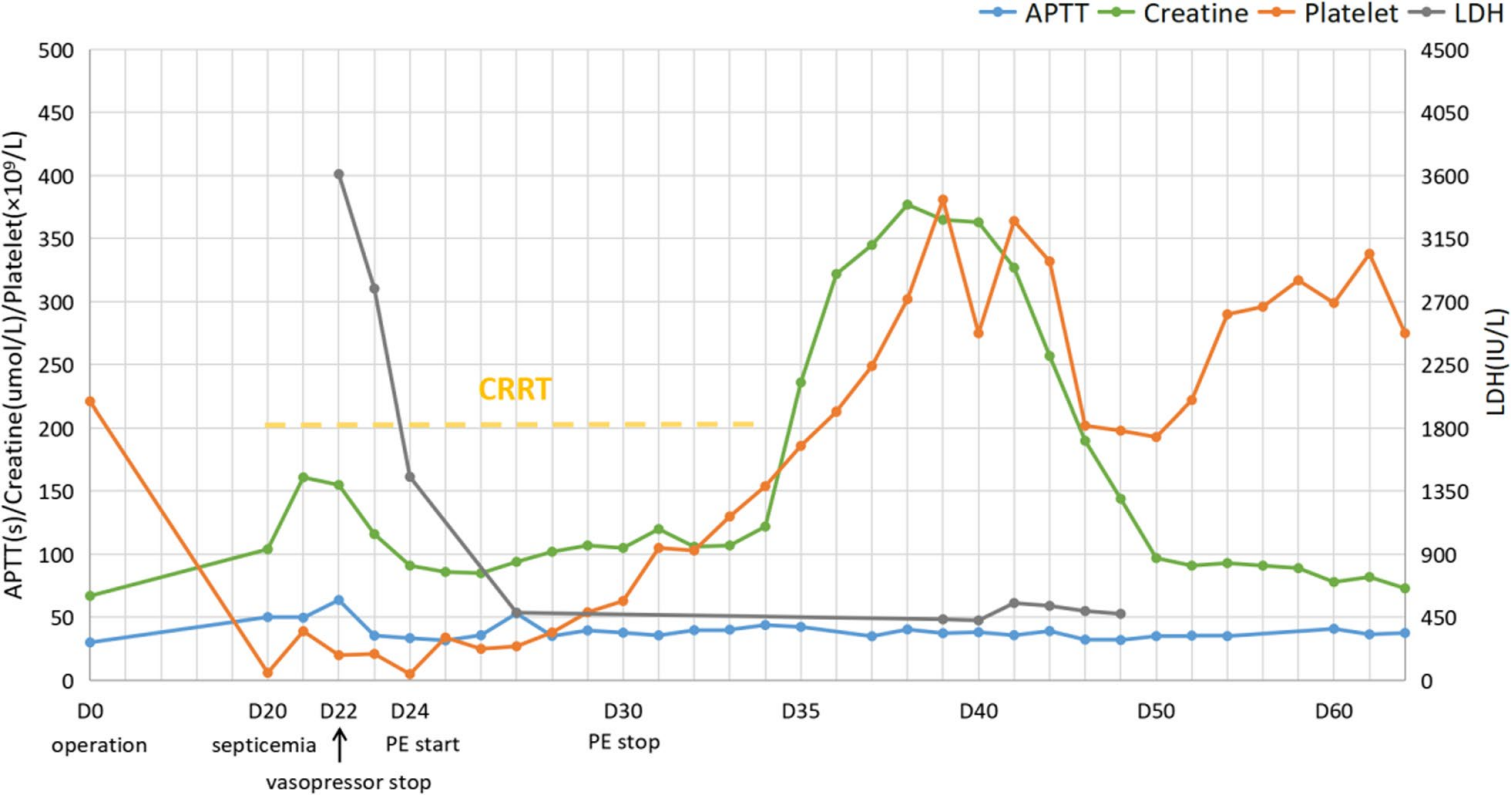
Timeline of clinical manifestation, laboratory parameters and treatment. *D* postoperative day, *LDH* lactate dehydrogenase, *APTT* activated partial thromboplastin time, *PE* plasma exchange, *CRRT* continuous renal replacement therapy

Her overt DIC score was 6, and her sepsis-induced coagulopathy score was 6. A diagnosis of septic shock due to Salmonella derby, with complications of DIC and TMA, was established. The Antibiotic was adjusted from ceftriaxone to meropenem on the arrival day to ICU. She received fluid resuscitation, vasopressor, mechanical ventilation, dialysis, and hydrocortisone for septic shock (200–300 mg/d increase from 100 mg/d). She received transfusions of 10.5U packed red blood cells, 800 ml fresh frozen plasma, and four packs of platelets from the 21st to the 23rd postoperative day. On the 24th postoperative day, plasma exchange therapy (fresh frozen plasma 2–2.4 L/d) started immediately. She underwent seven consecutive plasma exchange therapies in total (Fig. 1).

She made a rather good recovery, fortunately. She was extubated on the 31st postoperative day. As she regained her platelet count of 63 × 10^9^/L on the 32nd postoperative day, plasma exchange therapy ceased. Continuous veno-venous hemodialysis stopped the next day as she was able to urinate 868 ml/day. Her plasma ADAMTS13 activity raised to 86% (Table 2). During the whole course of the illness, her neurological status stayed alert. She was discharged from ICU on the 38th postoperative day with renal dysfunction (urea nitrogen, 24.07 mmol/L; creatinine, 344 umol/L).

She was discharged from the hospital with normal renal function (urea nitrogen, 7.17 mmol/L; creatinine, 73 umol/L) on the 68th postoperative day.

## Discussion and conclusions

Salmonella derby was one serovar out of over 2500 recognized serovars of Salmonella causing invasive non-typhoidal salmonella infections(iNTS), presenting as septicemia with and without a secondary extra-intestinal focus of infection. iNTS has a lower prevalence yet a greater severity and case fatality than Salmonella enteritidis or typhoid fever [21]. Salmonella derby was one of the top five causes of iNTS in China [22, 23]. At the same time, it rated behind 30 in the United States [24]. Corticosteroid use increases the risk of non-typhoidal salmonella septicemia [25, 26].

Our case highlights one significant finding: an uncommon secondary TMA in the context of Salmonella septicemia-induced DIC. It is the first report of the co-occurrence of DIC and TMA in Salmonella septicemia.

Most cases of Salmonella septicemia have been diagnosed with DIC based on the overt DIC criteria [27]. Our patient fulfilled both overt DIC (score 6) and sepsis-induced coagulopathy criteria (score 6) for diagnosis of DIC [27, 28]. She manifested with cutaneous and mucosal bleeding and subcutaneous hematoma, abnormal coagulation, thrombocytopenia, elevated FRMs, and multiorgan dysfunction. However, later during her resolution of sepsis, the elevation of indirect bilirubin, and persistent severe anemia, which was not consistent with the severity of bleeding, indicated hemolytic anemia. The increased LDH and reticulocyte percentage, with peripheral schistocytes, demonstrated MAHA. Measurement of haptoglobin is not available in our hospital. MAHA also presents in DIC, but TMA is a more appropriate diagnosis when atypical MAHA appears. ADAMTS13 activity (before initiation of plasma exchange therapy), microbiological testing, and complement factor testing helped us rule out TTP, STEC-HUS, and aHUS. Our patient was diagnosed with an infection-associated TMA. Thereby co-occurrence of DIC and TMA complicated septicemia had been finally established. Salmonella septicemia triggered overt DIC and TMA simultaneously and incidentally from clues in the timeline of coagulation and hemolysis.

DIC and TMA occasionally co-occurred in previous literature [1, 29]. Most cases were patients with sepsis as Entero-hemorrhagic Escherichia coli, Proteus mirabilis, Group A Streptococcus, and Capnocytophaga canimorsus [1, 30–37]. Other cases were patients with bone marrow metastasis from gastric cancer and liver failure [1, 38]. From the Oklahoma Thrombotic Thrombocytopenic Purpura-Hemolytic Uremic Syndrome registry, 32.3% (10/31) of patients with TTP manifested coagulation disorders, indicating DIC development [39]. Up to 14% of patients in one STEC-HUS cohort experienced DIC during the disease [40]. Multicentric studies are needed to determine the actual risk of co-occurrence of DIC and TMA.

It remains unclear whether these were the co-morbid state of DIC and TMA or TMA progression mediated by coagulopathy due to DIC [15] or, in fact, misdiagnosed cases. These findings propose that TMA should not be overlooked in patients with DIC. Signs of MAHA and thrombocytopenia would be a critical clue for TMA diagnosis. In suspicious TMA cases in the context of DIC, further investigations of Shiga-toxins, complements, ADAMTS13 activity, and inhibitors are recommended [11]. Renal biopsy, if feasible, also played a vital part in TMA diagnosis. Detection of microvascular thrombosis, especially platelet thrombosis on biopsy, usually indicates TMA. Fibrinolysis may dissolve micro-thrombosis in patients with DIC [1]. As classic pathological entities in TMA and DIC have been identified, renal biopsy contributes to better differentiation [15]. Nevertheless, our patient had rejected renal biopsy.

Regarding treatment, the keystone in managing DIC and TMA is the adequate treatment of the etiology. In the present case, it would be the antibiotics targeting Salmonella septicemia.

In DIC, platelet transfusion is advised in patients with bleeding tendencies due to thrombocytopenia [41, 42]. Anti-coagulation or anti-fibrinolytic therapy is indicated according to DIC’s hypercoagulative or hyperfibrinolytic state [41, 42]. Antithrombin concentrate and recombinant thrombomodulin for DIC are frequently used in Japan [43, 44].On the contrary, platelet transfusion is contraindicated in TMA [1]. In our case, recognition of TMA prevented the patient from deterioration as we stopped further transfusion of platelet, which might be the reason for persistent MAHA and thrombocytopenia.

Different therapeutic intervention according to specific etiology is advised. Plasma exchange therapy is recommended in TTP, eculizumab is useful for aHUS, and rituximab is effective for acquired TTP [45–47]. Plasma exchange therapy is necessary to start empirically in adult patients with TMA of unclear etiology to avoid delaying TTP treatment [11, 13, 48, 49]. Plasma exchange therapy in TMA is considered helpful in replenishing ADAMTS13, eliminating antibodies to ADAMTS13, replacing normal-sized von Willebrand factor (vWF), and eliminating ultra vWF multimers and excess cytokines. Plasma exchange therapy also had a role in sepsis or septic shock with DIC, TMA, and multiple organ dysfunction [50–53]. Plasma exchange therapy also benefited septic patients with decreased ADAMTS13 activity [54]. The possible underlying mechanism of plasma exchange therapy was via repairing endothelial function in sepsis and DIC. Evidence for therapeutic interventions beneficial in sepsis complicated with DIC and TMA is lacking. Therapeutic interventions were adopted empirically and varied from case to case in previous reports. A specific treatment according to the etiology of TMA could be a reasonable strategy for a better outcome.

However, the relationship between ADAMTS13 activity and severity, organ failure and outcome in sepsis, and sepsis-associated DIC remained controversial [37, 55–58]. One hypothesis is that severe endothelial injury in sepsis-associated TMA leads to the release of a massive number of vWF multimers and the consumption of ADAMTS13, contributing to a mild decrease in ADAMTS13 activity [59]. Another hypothesis is that damage to endothelium and activation of the complement system in systemic inflammation in sepsis and DIC followed by the formation of neutrophil extracellular traps, which affects the change of ADAMTS13 structure and reduces the activity of ADAMTS13 [59–61]. Both endothelial injuries and neutrophil extracellular traps might contribute to the pathogenesis of the co-occurrence of TMA and DIC.

In conclusion, constant vigilance is necessary to avoid missing TMA in patients with sepsis-associated DIC. DIC itself should not be an exclusion for TMA diagnosis. An atypical clinical course for sepsis-associated DIC with atypical MAHA and thrombocytopenia often implies the clue for TMA. A throughout workup, including microbiological testing, ADAMTS13 activity, and complement factor testing, is necessary for the diagnosis of TMA etiologies. Proper and timely recognition of TMA is vital for appropriate decisions regarding the use of plasma exchange therapy and restriction of platelet transfusion in patients with co-occurrence of DIC and TMA.

## Supplementary Information

**Additional file 1:** Laboratory workup for thrombotic microangiopathy.

## Data Availability

All data included in this case are included in this published article. All data are available from the corresponding author upon reasonable request.

## Abbreviations

DIC: Disseminated intravascular coagulation
TMA: Thrombotic microangiopathy
FRMs: Fibrin-related markers
ICU: Intensive care unit
MAHA: Microangiopathic hemolytic anemia
STEC-HUS: Shiga toxin-associated hemolytic uremic syndrome
aHUS: Atypical hemolytic uremic syndrome
TTP: Thrombotic thrombocytopenic purpura
ADAMTS13: A disintegrin-like and metalloprotease with thrombospondin type 1 motifs 13
AKI: Acute kidney injury
FDPs: Fibrinogen and fibrin degradation products
LDH: Lactate dehydrogenase
iNTS: Invasive non-typhoidal salmonella infections
vWF: von Willebrand factor
