# Supplementary material for: Case report of Salmonella derby septicemia complicated with co-occurrence of disseminated intravascular coagulation and thrombotic microangiopathy
Source: BMC Infect Dis. 2022 Dec 7;22:914. doi: 10.1186/s12879-022-07913-2 (PMC9730593; doi:10.1186/s12879-022-07913-2)
Supplement: Supplementary file 1 — Additional file 1: Laboratory workup for thrombotic microangiopathy. [file 12879_2022_7913_MOESM1_ESM.docx]

Additional file 1: Table S1. Laboratory workup for thrombotic microangiopathy

| Laboratory test | result | Normal range |
| --- | --- | --- |
| complement3 | 0.83 | 0.70–1.40 g/L |
| complement4 | 0.11 | 0.10–0.40 g/L |
| CH50 | 30.3 | 23.0–46.0 U/mL |
| anticardiolipin antibody IgM | 2.05 | <8.0 MPLU/ml |
| anticardiolipin antibody IgG | 3.94 | <8.0 GPLU/ml |
| β2 glycoprotein antibody IgM | 3.37 | 0–20 AU/ml |
| β2 glycoprotein antibody IgG | 3.04 | 0–20 AU/ml |
| IgM | 0.89 | 0.50–2.80g/L |
| IgG | 6.68 | 8.60–17.40g/L |
| IgA | 1.61 | 1.00–4.20g/L |
| anti-nuclear antibody | negative | <1:100 negative |
| anti-double-stranded DNA | negative | negative |
| SS-A antibody | negative | negative |
| SS-B antibody | negative | negative |
| anti-Histone antibody | negative | negative |
| anti-mitochondrial antibody M2 | negative | negative |
| anticentromere antibody | negative | negative |
| anti-ribosomal P protein antibody | negative | negative |
| anti-nucleosome antibody | negative | negative |
| anti-proliferating cell nuclear antigen antibody | negative | negative |
| nRNP/Sm antibody | negative | negative |
| anti-Smith antibody | negative | negative |
| anti-Scleroderma 70 Antibody | negative | negative |
| anti RO52 | negative | negative |
| anti-polymyositis scleroderma antibody | negative | negative |
| anti-Jo-1 | negative | negative |
| MPO antibody | negative | negative |
| proteinase antibody | negative | negative |
| glomerular basement membrane antibody | negative | negative |
| perinuclear ANCA | negative | negative |
| cytoplasmic ANCA | negative | negative |
| ferritin | 347.8 | 4.63–204.0ng/mL |
| vitamin B12 | 1031.90 | 189.0–883.0pg/mL |
| folic acid | 5.86 | 2.7–34.0ng/mL |
| RBC CD 59+ | 100% | >95.00% |
| WBC CD59+ | 99.81% | >95.00% |
| direct Coombs’ test | Negative | Negative |
| indirect Coombs’ test | Negative | Negative |
| G6PD activity | 1.5 | 1.0–2.6 |
| anti-platelet autoantibody IgM | 3.84 | <5% |
| anti-platelet autoantibody IgG | 0.64 | <5% |
| anti-platelet autoantibody IgD | 0.14 | <5% |
| anti-platelet autoantibody IgA | 0.30 | <5% |
| ADAMTS13 activity (ICU day5) | 46% | >70% |
| ADAMTS13 activity (ICU day14) | 86% | >70% |
| ADAMTS13 inhibitor | Negative | Negative |
| complement factor H | Negative | Negative |
| complement factor H antibody | Negative | Negative |

CH50, fifty percent hemolytic unit of complement; IgM, immunoglobulins M; IgG, immunoglobulins G; IgA, immunoglobulins A; IgD, immunoglobulins D; ANCA, anti-neutrophil cytoplasmic antibody; MPO, myeloperoxidase; ADAMTS13, plasma A disintegrin-like and metalloprotease with thrombospondin type 1 motifs 13.
